# Comparative Proteomic Analysis of Fucosylated Glycoproteins Produced by *Bacteroides thetaiotaomicron* Under Different Polysaccharide Nutrition Conditions

**DOI:** 10.3389/fmicb.2022.826942

**Published:** 2022-03-04

**Authors:** Xiao Tian, Hao Jiang, Binbin Cai, Huxin Feng, Xuan Wang, Guangli Yu

**Affiliations:** ^1^Key Laboratory of Marine Drugs of Ministry of Education, Shandong Provincial Key Laboratory of Glycoscience and Glycotechnology, School of Medicine and Pharmacy, Ocean University of China, Qingdao, China; ^2^Laboratory for Marine Drugs and Bioproducts, Pilot National Laboratory for Marine Science and Technology, Qingdao, China

**Keywords:** *Bacteroides thetaiotaomicron*, fucosylation, proteomics, metabolic glycan labeling, polysaccharide

## Abstract

Bacteroides *thetaiotaomicron*, one of the most eminent representative gut commensal *Bacteroides* species, is able to use the *L*-fucose in host-derived and dietary polysaccharides to modify its capsular polysaccharides and glycoproteins through a mammalian-like salvage metabolic pathway. This process is essential for the colonization of the bacteria and for symbiosis with the host. However, despite the importance of fucosylated proteins (FGPs) in *B. thetaiotaomicron*, their types, distribution, and functions remain unclear. In this study, the effects of different polysaccharide (corn starch, mucin, and fucoidan) nutrition conditions on newly synthesized FGPs expressions and fucosylation are investigated using a chemical biological method based on metabolic labeling and bioorthogonal reaction. According to the results of label-free quantification, 559 FGPs (205 downregulated and 354 upregulated) are affected by the dietary conditions. Of these differentially expressed proteins, 65 proteins show extremely sensitive to polysaccharide nutrition conditions (FGPs fold change/global protein fold change ≥2.0 or ≤0.5). Specifically, the fucosylation of the chondroitin sulfate ABC enzyme, Sus proteins, and cationic efflux system proteins varies significantly upon the addition of mucin, corn starch, or fucoidan. Moreover, these polysaccharides can trigger an appreciable increase in the fucosylation level of the two-component system and ammonium transport proteins. These results highlight the efficiency of the combined metabolic glycan labeling and bio-orthogonal reaction in enriching the intestinal *Bacteroides* glycoproteins. Moreover, it emphasizes the sensitivity of *Bacteroides* fucosylation to polysaccharide nutrition conditions, which allows for the regulation of bacterial growth.

## Introduction

*Bacteroides* are the most abundant Gram-negative obligate anaerobic bacteria in the distal small intestine and colon of mammals (Moore and Holdeman, 1974; Ushijima et al., 1983; Oren et al., 2016). In fact, each gram of human feces contains 10^10^–10^11^ *Bacteroides* cells (Flint et al., 2008). Considering the essential role of *Bacteroides* in maintaining human health and in gut microbe–host symbiosis, these species have attracted considerable attention. Specifically, *Bacteroides thetaiotaomicron*, a representative gut commensal *Bacteroides* frequently detected in the feces of healthy adults, has been thoroughly investigated (Xu et al., 2003). Despite being an opportunistic pathogen, *B. thetaiotaomicron* normally forms a harmonious and mutually beneficial symbiotic relationship with the host during its long-term evolution (Brook et al., 2013; D’Argenio and Salvatore, 2015). In particular, *B. thetaiotaomicron* helps the host degrade complex carbohydrates (Donaldson et al., 2016), suppress obesity (Ley et al., 2006), ameliorate colon inflammation (Delday et al., 2019), regulate colonic neuronal innervation and neurogenic function (Aktar et al., 2020), and even resist the invasion of pathogens (Xu and Gordon, 2003).

As an important component of cell surface glycoconjugates in gut microbes and host intestinal mucosa, *L*-fucose plays an important role in *Bacteroides* colonization and in gut microbe-host symbiosis (Pickard and Chervonsky, 2015; Garber et al., 2021) and it mainly exists in the form of an *O*-glycosylation protein (Martens et al., 2008; Fletcher et al., 2009; González-Morelo et al., 2020). There are two pathways in *Bacteroides* for biosynthesizing GDP-fucose, the donor for fucosyltransferase-catalyzed incorporation of fucose into bacterial capsular polysaccharides and fucosylated proteins (FGPs). One is the novo pathway, which is characterized by conversion of GDP-mannose to GDP-fucose by GDP-mannose dehydratase and GDP-fucose synthetase. The other one is the salvage pathway, in which exogenous fucose also can be transformed to GDP-fucose by Fkp, a bifunctional enzyme with both fucokinase and pyrophosphorylase activity (Coyne et al., 2005). Among different gut *Bacteroides*, *B. thetaiotaomicron* can stimulate the host to increase the α-1,2 fucosylation level through a series of cascade reactions, which inhibits the growth of harmful bacteria and benefits the growth of symbiotic bacteria when the host is in sickness (Goto et al., 2014; Pickard et al., 2014; Kostopoulos et al., 2021). Therefore, fucosylation is an important factor for the harmonious symbiosis between *Bacteroides* and the host. As the main product of fucosylation, FGPs may play critical roles in this process.

Compared to other *Bacteroides* species, *B. thetaiotaomicron* can more efficiently degrade various types of polysaccharides that are ultimately used to supply carbon and energy to the bacteria. The type strain VPI-5482 has 88 polysaccharide utilization loci (PULs) which cover 18% of its genome, and it encodes the proteins involved in sensing, importing, degrading, and regulating dietary carbohydrates in the colonic ecosystem (Xu et al., 2003; Martens et al., 2008, 2009; Rogers et al., 2013). Interestingly, PULs are often implicated with the ECF-type sigma factor in an environment response mechanism that co-regulates the ability of the bacteria to utilize nutrition (Donaldson et al., 2016). For example, starch is easily degraded by *B. thetaiotaomicron via* the starch utilization system (Sus) that includes homologues of SusC, SusD, and various carbohydrate-active enzymes (CAZymes) (Foley et al., 2018; Stevenson et al., 2021). In addition to starch, these homologs and enzymes can break down a variety of polysaccharides that are supplied by the diet or generated by the host (El Kaoutari et al., 2013). Similarly, fucoidan, a complex sulfated polysaccharide, cannot be digested by the human intestine without the help of intestinal microbiota (Morya et al., 2012). Previous studies have shown that fucoidan in *Ascophyllum nodosum*, *Laminaria japonica*, and sea cucumbers increases the content of intestinal *Bacteroides* (Shang et al., 2018; Hu et al., 2019; Li et al., 2019). Moreover, when dietary carbohydrates are deficient (e.g., during fasting or in the case of low-fiber diets), *B. thetaiotaomicron* changes its transcriptional profile in order to produce multiple fucosidases that cleave fucose from the intestinal mucin of the host, which increases the level of fucose in the gut lumen (Koropatkin et al., 2009). The cleaved fucose is then utilized by gut microorganisms. For example, *B. thetaiotaomicron* transforms fucose into glycoproteins, which promotes the adaptation of the bacterial species to the intestinal environment (Sonnenburg et al., 2005). The ability of *B. thetaiotaomicron* to synthesize its own fucose glycoconjugates using host-derived and dietary polysaccharides raises questions regarding the response of FGPs to nutrient variations and the functional roles of these FGPs in mediating a symbiotic relationship with the host.

The traditional strategies for glycoprotein enrichment, including the methods based on lectins and antibodies, have been successfully used to characterize prokaryotic glycoproteomes (Gonzalez-Zamnorano et al., 2009; Fredriksen et al., 2013; Leon et al., 2015). For example, Comstock and co-workers (Fletcher et al., 2009, 2011) determined 20 proteins in *B. fragilis* that were fucosylated using Aleuria aurantia lectin (AAL). The confirmed glycoproteins have putative roles in crucial cellular processes including cell division, chromosomal segregation, signaling processes, chaperone functions, protein-protein interactions, and peptidase/protease reactions (Fletcher et al., 2011). However, despite the efficiency of lectin- and antibody-based methods, they are not free of limitations. Most importantly, these methods are limited by the inability to detect newly glycosylated proteins in response to external stimulation (Longwell and Dube, 2013; Parker and Pratt, 2020). This is due to the fact that lectins label all of the existing glycoproteins that bear their glycan ligands. In the recent years, a powerful strategy for discovering new glycoproteins in mammals and bacteria has been proposed (Hong et al., 2019; Inoue et al., 2019; Zhou et al., 2019; Pedowitz et al., 2020). This strategy is based on the combination of metabolic oligosaccharide engineering (MOE) (Dube and Bertozzi, 2003) and bioorthogonal reactions, such as Cu(I)-catalyzed azide-alkyne cycloaddition (CuAAC) (Kolb et al., 2001; Sletten and Bertozzi, 2009; Neumann et al., 2020). Briefly, the strategy entails the incorporation of an unnatural monosaccharide with an active group into the specific glycan *via* the biological salvage metabolic pathway. The incorporated monosaccharide is then reacted with a probe comprising complementary and reporter groups through a bio-orthogonal reaction. This allows for the labeling of specific glycoproteins that can thus, be selectively analyzed using a variety of techniques, such as imaging, gel-based assays, and mass spectrometry (MS) (Riley et al., 2021). This strategy has been successfully applied in the identification and quantification of newly synthesized glycoproteins in many systems, including hippocampal synapses, cardiac hypertrophy of live mice, secretome of activated Jurkat cells, and even gut bacteria (Liu et al., 2009; Champasa et al., 2013; Rong et al., 2014; Schanzenbächer et al., 2016; Witzke et al., 2017). MOE and bioorthogonal reaction have also been used to label glycoconjugates in various symbiotic anaerobic bacteria, including *B. fragilis*, to describe the distribution and colonization of these bacteria along the intestine (Besanceney-Webler et al., 2011; Geva-Zatorsky et al., 2015).

To the best of our knowledge, the FGPs in *B. thetaiotaomicron* have not yet been identified, and their types, distribution, and functions remain unknown.

In this study, we utilized MOE and CuAAC to selectively label, enrich and identify the FGPs in *B. thetaiotaomicron* for the first time. The effects of different nutritional conditions on FGPs expression patterns are also determined by coupling metabolic glycan labeling and label-free quantification. The results could help to preliminarily clarify the underlying molecular biological mechanisms of FGPs in promoting intestinal colonization and mediating the symbiotic relationship with the host.

## Materials and Methods

### Metabolic Labeling of Fucosylated Glycan in *Bacteroides thetaiotaomicron*

*Bacteroides thetaiotaomicron* VPI-5482 (purchased from China General Microbiological Culture Collection Center) was incubated under anaerobic conditions at 37°C for 24 h in modified anaerobic minimal medium (AMM) (Varel and Bryant, 1974; Baughn and Malamy, 2002) with or without 200 μM alkynyl-fucose (FucAl) [synthesized as previously described (Schneider et al., 2018)], then the cells were collected for flow cytometry or FGPs enrichment. For the comparative proteomic analysis of FGPs under different carbohydrate nutritional conditions, *B. thetaiotaomicron* VPI-5482 was incubated in AMM containing 0.5% glucose (Sigama, United States) in anaerobic conditions at 37 °C for about 24 h and grew to the middle logarithmic growth phase (*OD*_600 *nm*_ = 0.5). The bacterial culture medium was divided into four groups: G (glucose), M (mucin), S (starch), and F (fucoidan). G group is the control, and no additional carbohydrate was added to this group. Mucin from porcine stomach (type II) (Sigma, St. Louis, MO, United States), corn starch (Solarbio, China) and fucoidan from *L. japonica* (Shandong Jiejing, China) were added to the other three corresponding groups to a final concentration of 0.25% (w/v), respectively. 200 μM FucAl was added at the same time. Cells were collected after culturing under anaerobic conditions at 37°C for 3 h. All groups mentioned above were done in three replicates.

### Labeling of Fucosylated Proteins With Biotinylated Probes *via* Cu(I)-Catalyzed Azide-Alkyne Cycloaddition in Cell Lysates

*Bacteroides thetaiotaomicron* cells were harvested by centrifugation (5,000*g*, 10 min), washed two times with PBS and resuspended in lysis buffer (1% NP-40, 150 mM NaCl, Roche protease inhibitor, 100 mM sodium phosphate pH 7.5). Cells were intermittently vortexed and sonicated for 20 min at 4°C. The supernatant was obtained by centrifugation (12,000*g*, 10 min, 4°C) and the protein concentration was determined by BCA protein assay kit (Thermo Fisher Scientific, Waltham, MA, United States). Then cell lysates were diluted to 2 mg/mL and reacted with 100 μM biotin-picolyl-azide (Click Chemistry Tools, Scottsdale, AZ, United States) in the reaction solution containing premixed BTTAA (Click Chemistry Tools, Scottsdale, AZ, United States)-CuSO_4_ complex ([BTTAA]: [CuSO_4_] = 1 mM: 0.5 mM) and 2.5 mM freshly prepared sodium ascorbate for 1.5 h with agitation (25 °C, 1200 rpm).

### Streptavidin Enrichment of Biotinylated Proteins

The biotin-labeled lysates were precipitated by 8 volumes of methanol at -40°C overnight. The precipitated proteins were collected by centrifugation at 5,000 *g* for 15 min at -10°C, washed with cold methanol for three times and resolubilized with 1.2% SDS/PBS. The solubilized proteins were incubated with streptavidin agarose resin (100 μL of slurry, Thermo Fisher Scientific, Waltham, MA, United States) in 0.2% SDS/PBS at RT for 3 h with rotation. After washing the beads with 0.2% SDS/PBS for one time, PBS for three times, and ddH_2_O for three times sequentially, biotinylated proteins were eluted from the beads by treating with 2 × SDS PAGE loading buffer in the presence of 2 mg/mL biotin for 10 min at 95°C to avoid streptavidin contamination (Cheah and Yamada, 2017). Excess biotin was removed by ultrafiltration.

### Flow Cytometry

*Bacteroides thetaiotaomicron* cells were harvested and resuspended in cold PBS. For cells treated with CuAAC, 100 μL reactions were setup containing 5 × 10^7^ cells, 100 μM biotin-picolyl-azide, 2.5 mM sodium ascorbate and BTTAA-CuSO_4_ complex ([BTTAA]:[CuSO_4_] = 360 μM: 60 μM). After 10 min reaction the cells were washed 3× with cold PBS and incubated in 1 μg/mL Streptavidin Alexa Fluor 488 conjugate (Thermo Fisher Scientific, Waltham, MA, United States) at 4°C for 30 min. Then the cells were washed 3× in cold PBS and applied to ACEA NovoCyte benchtop flow cytometer (Acea Biosciences, San Diego, CA, United States) using a 488 nm argon laser. Flow cytometry data were analyzed using NovoExpress.

### Verification of Fucosylation of Candidate Proteins

*Escherichia coli* S17-1λpir chemically competent cells containing the Rp4-2 plasmid (Beijing Zoman Biotechnology, China) were used as the donor for mobilization of plasmids to *Bacteroides* strains. *E. coli-Bacteroides* shuttle expression plasmid pFD340 (Whitehead and Hespell, 1990) was a gift from Dr. Smith and Dr. Rocha (East Carolina University). A 3.9 kb fragment of Q8A818, containing the coding regions and 1500 bp of upstream and 18 bp of 6× His tag gene at the C terminus was amplified from the *B. thetaiotaomicron* genome, and ligated to pFD340. Similarly, A 4.3 kb fragment of Q8A0P4 was amplified and ligated to BamHI and NarI digested pFD340. All primers were designed using Primer Premier and shown below. Underlined nucleotides indicate engineered restriction sites, and lowercase letters represent homology arm sequences.

Q8A818
F: 5′-agaattgactctagaggatccTCGTTTTTGGGGACGTT TGT-3′
R1: 5′-TTAATGGTGATGGTGATGATGTTTCTTTATAAG ATTAGCAATAGAAGCA-3′
R2: 5′-gaaaataccgcatcaggcgccTTAATGGTGATGGTGATG ATGTTTC-3′
Q8A0P4
F: 5′-agaattgactctagaggatccTTCTTATCATGTTGCATAA ATGCG-3′
R: 5′-gaaaataccgcatcaggcgccTTAATGGTGATGGTGATGA TGGAAACGCATAGGTCTGC-3′

Subclones were obtained after transformation of *E. coli* S17-1λpir chemically competent cells (containing the Rp4-2 plasmid) with the ligation mixtures. The resulting plasmids, pFD340-Q8A818 and pFD340-Q8A0P4, were mobilized into *B. thetaiotaomicron* by *E. coli*-to*-Bacteroides* matings as described previously (Shoemaker et al., 1985; Strand et al., 2014; Liou et al., 2020). Recombinant *B. thetaiotaomicron* was incubated in brain–heart infusion medium (BHI) (Thompson and Malamy, 1990) with and without 200 μL FucAl (containing 10 μg/mL Erm*^r^*) under anaerobic conditions at 37°C to the logarithmic growth phase of bacteria. Cell lysates were prepared and reacted with biotin-picolyl-azide *via* CuAAC, followed by streptavidin enrichment as described above.

### Western Blot Analysis

Proteins separated by SDS-PAGE were transferred to PVDF membrane. The membrane was incubated with blocking buffer [5% non-fat milk in TBST (Tris buffered saline with 0.1% Tween-20, pH 7.5)] for 1 h at room temperature (RT). The blocked membrane was incubated with HRP-anti-biotin antibody (Jackson ImmunoResearch, West Grove, PA, United States) (1:100,000) or an HRP-anti-His (27E8) antibody (Cell Signaling Technology, Danvers, MA, United States) (1:1000) in blocking buffer overnight at 4°C, washed three times with TBST and visualized with ECL Western blotting detection reagents (Thermo Fisher Scientific, Waltham, MA, United States). Odyssey FC imaging system (LI-COR, United States) was used to detect the chemiluminescence. For Aleuria Aurantia Lectin (AAL) blot, the membrane was blocked in 5% bovine serum albumin (BSA) (Sigma, St. Louis, MO, United States) for 1 h at RT and incubated with biotinylated-AAL (Vector Laboratories, Burlingame, CA, United States) (1:500) in PBS for 1 h at RT. After being washed for three times with TBST, the membrane was incubated with HRP-anti-biotin antibody and visualized.

### Label-Free Comparative Proteomic Quantitative Analysis

The total protein or enriched FGPs of various groups were reduced with 5 mM TCEP for 30 min at 37°C, and alkylated with 15 mM IAA for 30 min in the dark at RT. Each sample was trypsinized by the filter aided proteome preparation (FASP) method. The peptides were desalted by C18 Cartridge, lyophilized and reconstituted by 40 μL of 0.1% formic acid solution.

Peptides were submitted to label-free differential proteomic analysis. Each sample was separated with a nanoliter flow rate HPLC liquid system Easy nLC. The mobile phases consisted of a 0.1% (v/v) formic acid aqueous solution (A) and 0.1% (v/v) formic acid in acetonitrile/water (84% acetonitrile) (B). The column was equilibrated with 95% solution A. Samples were loaded from the autosampler to the loading column (Thermo Scientific Acclaim PepMap100, 100 μm × 2 cm, nanoViper C18), and passed through the analytical column (Thermo scientific EASY column, 75 μm × 10 cm, C18-A2), with a flow rate of 300 nL/min. Samples were chromatographed and analyzed by Q-Exactive mass spectrometer. Detection parameters were used as follows: detection method (positive ion); scanning range of the precursor ion (300–1800 m/z); resolution of the first-order mass spectrum (70,000 at 200 m/z); automatic gain control target (1e^6^); maximum IT (50 ms); dynamic exclusion time (60.0 s). Mass-to-charge ratios of peptides and peptide fragments were collected according to the following methods: fragment maps taken after each full scan (20); MS2 activation type (HCD); isolation window (2 m/z); MS2 resolution (17,500 at 200 m/z); normalized collision energy (30 eV); underfill (0.1%).

MaxQuant software (version 1.5.3.17) was used to the database searching against UniProt -*Bacteroides thetaiotaomicron* (strain ATCC 29148/DSM 2079/NCTC 10582/E50/VPI-5482) and FASTA database (2019_07 release, 4783 reviewed entries). Searching parameters were used as follows: max missed cleavages (2); fixed modification, carbamidomethyl (C); variable modifications, oxidation (M); MS/MS tolerance (20 ppm); protein and peptide FDR (≤0.01); peptides used for protein quantification (razor and unique peptides); time window (2 min); protein quantification (LFQ/iBAQ). Groups M, S, and F were compared with group G, and the statistical analysis of T test and FDR (Benjamini-Hochberg) were performed. A fold change cut-off of ≥2 with *p*-value < 0.05 was used as threshold to define significantly changed proteins.

### Bioinformatic Analysis of Protein Identifications

Blast2Go was used to annotate the GO functions of the FGPs. GO function enrichment analysis was performed by Fisher’s exact test method. KEGG was used to perform pathway-related analysis on the FGPs. The unit was KEGG pathway, and the total proteins identified were used as the background. Fisher’s exact test was used to analyze and calculate the significance level of protein enrichment in each pathway to determine the metabolic and signal transduction pathways that were significantly affected. In addition to the GO and KEGG annotation and enrichment analysis of the differential proteins according to the above methods, cluster analysis and protein-protein interaction of these proteins were also performed. The quantitative information of target proteins was normalized. The ComplexHeatmap R package (R Version 3.4) was used to classify the two dimensions of the sample and protein expression levels to generate a hierarchical clustering heatmap. The protein–protein interaction of differential proteins was conducted on the STRING database^1^ and Cytoscape software.

## Results

### Labeling and Enrichment of Fucosylated Proteins in *Bacteroides thetaiotaomicron*

**FIGURE 1:**
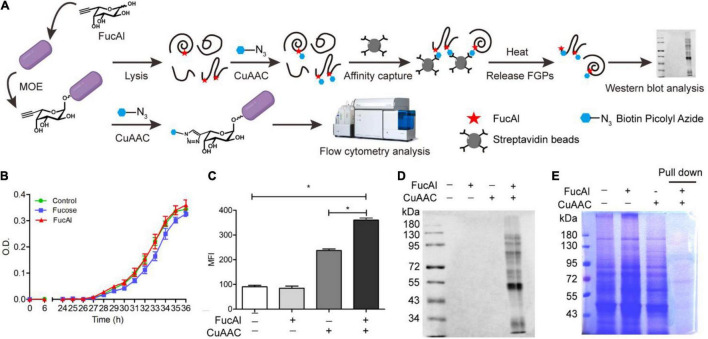
Detectionof *B. thetaiotaomicron’*s FGPs by metabolic labeling and CuAAC. **(A)** Workflow for labeling, enrichment and analysis of FGPs in *B. thetaiotaomicron*. **(B)** Evaluation of biocompatibility of FucAl in *B. thetaiotaomicron* by bacterial growth curve. **(C)** Flow cytometry analysis of labeling efficiency of FGPs on the cell surface of *B. thetaiotaomicron*. **(D)** Western blot analysis of labeling of *B. thetaiotaomicron’*s FGPs by HRP-anti-biotin antibody. **(E)** Coomassie brilliant blue staining of total proteins and the enriched FGPs. Errors were based on triplicate runs in one experiment. *P < 0.05.

The strategy adopted in this study for FGPs labeling in *B. thetaiotaomicron* is based on previous research (Besanceney-Webler et al., 2011). First, the fucose analog bearing a terminal alkynyl substituent at C-6 position, FucAl, was introduced into the culture media, and was metabolically incorporated into the glycoconjugates of *B. thetaiotaomicron*. Then, the alkyne-modified glycoproteins in the lysate solutions were chemically reacted with biotin-picolyl-azide *via* CuAAC, and then were enriched by streptavidin beads. This method allows for the assessment of the labeled FGPs using Western blot and flow cytometry analyses (Figure 1A). To evaluate the biocompatibility of FucAl, *B. thetaiotaomicron* was anaerobically cultured in minimal medium, in the presence or absence of 200 μM FucAl or fucose. The obtained growth curve shows that the addition of FucAl does not adversely affect the proliferation of *B. thetaiotaomicron*, even after 36 h, which suggests that it is biocompatible (Figure 1B). Moreover, based on the results of the Western blot and flow cytometry experiments, the added FucAl (200 μM) does not appreciably induce the hyperfucosylation of proteins and has no effect on the expression level of FGPs within 24 h. This confirms the biocompatibility of FucAl and indicates that it may be used to label FGPs (Supplementary Figure 1).

Considering that *B. thetaiotaomicron* and *B. fragilis* share a common fucose acquisition system (Xu et al., 2003; Coyne et al., 2005), it was assumed that FucAl can also be applied in the labeling of *B. thetaiotaomicron* glycoproteins. To verify the labeling of *B. thetaiotaomicron* FGPs by FucAl and CuAAC, the biotinylated glycoconjugates on the bacterial surface were analyzed by flow cytometry using the streptavidin-Alexa Fluor 488. As expected, the FucAl- and CuAAC-treated *B. thetaiotaomicron* exhibit high intensity fluorescence, whereas the untreated cells show minor fluorescence (Figure 1C). These biotinylated FGPs were also detected in the lysates by Western blotting with HRP-anti-biotin (Figure 1D). After that, biotinylated FGPs were almost completely captured by streptavidin beads and eluted effectively (Supplementary Figure 2), and based on SDS-PAGE analysis of the eluent, the enriched FGPs account for only a small portion of total proteins (Figure 1E).

### *Bacteroides thetaiotaomicron* Culturing and Fucosylated Proteins Profiling Under Different Polysaccharide Nutrition Conditions

**FIGURE 2:**
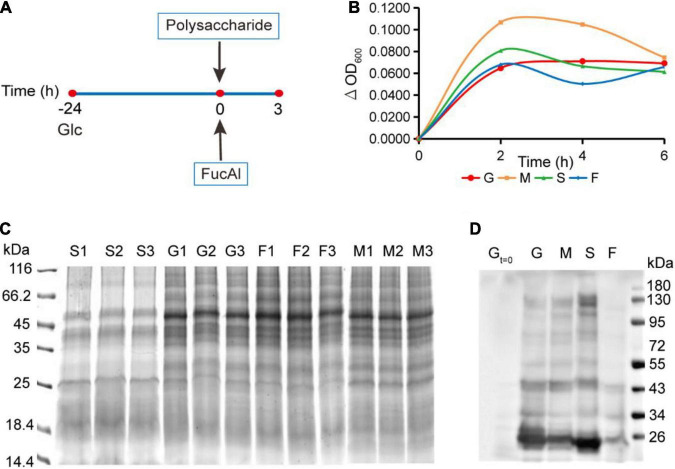
Culture of *B. thetaiotaomicron* and enrichment of FGPs in different polysaccharide nutrition conditions. **(A)** Scheme for culture of *B. thetaiotaomicron*. *B. thetaiotaomicron* was incubated in AMM (0.5% glucose) under anaerobic conditions at 37°C for 24 h and grew to the middle logarithmic growth phase. No additional carbohydrate was added to the G group. Mucin (M group), starch (S group), and fucoidan (F group) were added into the corresponding group to a final concentration of 0.25% (m/v), respectively. 200 μM FucAl was added at the same time. After 3 h, bacterial cultures were collected. **(B)** Growth curves of *B. thetaiotaomicron* under different polysaccharide nutrition conditions. G, glcucose; M, mucin; S, corn starch; F, fucoidan. **(C)** Gel imaging of global proteins in each experiment. **(D)** Western blot analysis of enriched FGPs in each group by anti-biotin antibody.

Considering the importance of FGPs in maintaining the growth and survival of *Bacteroides*, it is important to assess the effects of different polysaccharide nutrition conditions on the expression of newly synthesized FGPs in *B. thetaiotaomicron*. For this purpose, we next sought to apply FucAl as the probe to investigate it. *B. thetaiotaomicron* was cultured in a normal glucose medium up to the mid-logarithmic phase with a relatively strong metabolic state. Then, FucAl and different types of polysaccharides, including those derived from dietary land plants (corn starch), dietary marine algea (fucoidan), and the host (porcine mucin-type *O*-glycans), were added to the cultures (Figure 2A). FucAl was used to label the newly fucosylated proteins in *B. thetaiotaomicron* within 3 h. Actually, very little of these added polysaccharides was used as the carbon sourse by the bacteria in the short period of time. Consequently, in this case, *B. thetaiotaomicron* was triggered by the newly added polysaccharides, and the changes of newly synthesized FGPs were due to the response to the changes of nutrient conditions, rather than the utilization of these polysaccharides. Finally, the total proteins and FGPs in these cultures were separately collected. As shown in Figure 2B, bacterial growth reaches a maximum at almost the same time, irrespective of the polysaccharide nutrition condition, and group M presents the highest amount of bacteria at the end of the exponential phase. Based on SDS-PAGE analysis, the expression patterns of total protein are similar in all groups (Figure 2C). In contrast, the expression patterns of FGPs were significantly different according to the Western blot result (Figure 2D). This stimulated our interest to further explore the expression differences of FGPs *via* comparative proteomics.

### Fucosylated Proteins Identification by Label-Free Quantitative Mass Spectrometry Analysis

**FIGURE 3:**
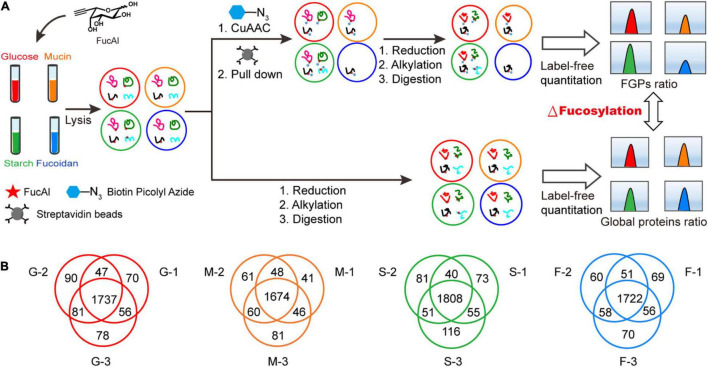
Label-free quantitative comprehensive proteomes of FGPs. **(A)** Workflow for label-free quantitative comprehensive proteomes of FGPs. **(B)** Venn diagrams of three repeated experiments in each group.

The above results indicate that the addition of exogenous polysaccharides has a greater impact on FGPs expression in *B. thetaiotaomicron* than on the level of total proteins. In order to identify the FGPs in response to different nutritional conditions, the methods of metabolic labeling and CuAAC with label-free comparative quantification were applied (Figure 3A). *B. thetaiotaomicron* was cultured as described in Figure 2A. At 3 h after polysaccharides treatment, bacterial cultures were collected, and the differences in expression patterns of total proteins and streptavidin enriched FGPs were both studied by label-free quantification (Figure 3A). The false discovery rate (FDR) for peptides and proteins was adjusted to less than 1%, and the mass deviations of all the identified peptides were mainly within 10 ppm. Each MS2 map presented an ideal Andromeda score, with 82.55% of the peptides scoring above 60 points. The median score of all the of the peptides was found to be 101.62 points. The peptide number distribution and peptide sequence coverage further confirm the high quality, accuracy, and reliability of the peptides identified herein (Supplementary Figure 3). The experiments were performed in triplicates, and the results indicate high reproducibility in terms of the identified FGPs (Figure 3B).

### Fucosylation Verification of Candidate Fucosylated Proteins

**FIGURE 4:**
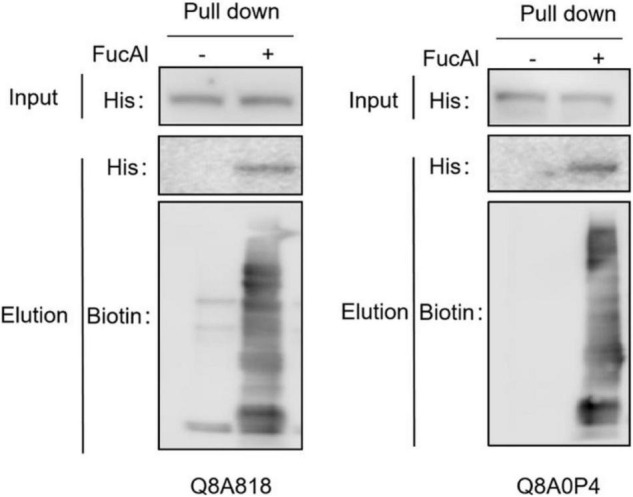
Fucosylation verification of two candidate FGPs. The cell lysates were prepared and reacted with biotin-picolyl-azide *via* CuAAC, followed by streptavidin beads pull-down. The enriched proteins were analyzed by Western blot with anti-His and anti-biotin antibody.

To verify the fucosylation of the identified FGPs, two proteins (Q8A818 and Q8A0P4) with high scores and LFQ intensities were selected as candidate compounds. Q8A818 is a capsular polysaccharide transport protein that can transport polysaccharides to the capsule of *B. thetaiotaomicron*, resulting in the formation of loose mucus substances on the surface of *Bacteroides* cell walls. These substances are essential for the survival of the bacteria (Hsieh et al., 2020). Q8A0P4 is a TonB-dependent outer membrane receptor that is mainly responsible for the precipitation of ferritin in Gram-negative bacteria and for the intake of various nutrients, such as iron complexes, vitamin B12, and carbohydrates. Studies have shown that this type of receptor acts as an adhesion molecule that binds to the host fibronectin, and that it is related to the pathogenicity of *Bacteroides* (Koebnik, 2005; Miethke and Marahiel, 2007; Schauer et al., 2008; Pauer et al., 2009). Recombinant plasmids containing 6 × His-tagged recombinant proteins were constructed by homologous recombination and transferred to *B. thetaiotaomicron* by *E. coli*-to-*Bacteroides* mating. The total FGPs in the bacteria were then labeled as described above and subjected to pull-down with anti-biotin antibody. The western blot results illustrated in Figure 4 show that both the control and alkyne-labeled samples present His-tagged protein signals, which suggests that the candidate proteins had been successfully expressed in *B. thetaiotaomicron*, and that the addition of FucAl did not affect their expression. Compared with control, the enriched FGPs in alkyne-labeled samples show obvious signals of anti-biotin and anti-His, which suggests that the total FGPs contained Q8A818 and Q8A0P4. All the above results confirmed that the detected Q8A818 and Q8A0P4 proteins were indeed fucosylated.

### Screening and Annotation of Differentially Expressed Fucosylated Proteins

**FIGURE 5:**
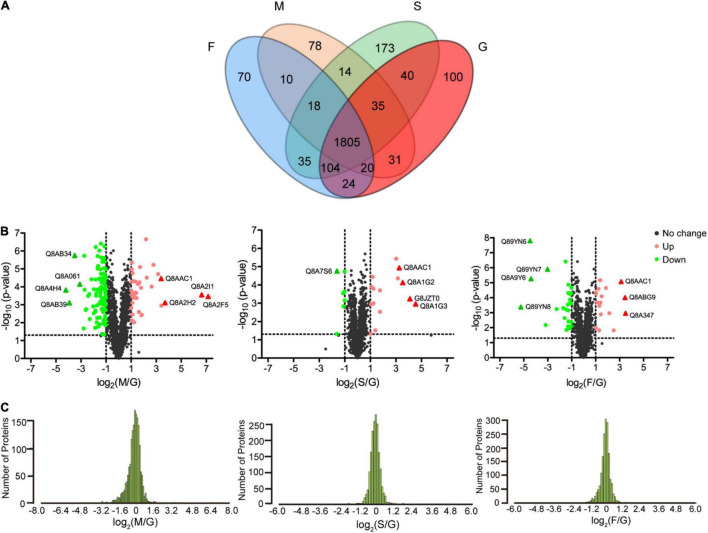
Analysis of differentially expressed FGPs in different comparisons. **(A)** Venn diagram of differentially expressed FGPs of *B. thetaiotaomicron* under four carbohydrate nutrition conditions. **(B)** Volcano plots depicting the enrichment (x axis) and significance (y axis) of the identified FGPs for mucin/glucose (left), starch/glucose (middle), and fucoidan/glucose (right). **(C)** Normal distribution of differentially expressed FGPs for mucin/glucose (left), starch/glucose (middle), and fucoidan/glucose (right).

According to the threshold for differentially expressed proteins (*p*-values < 0.05, fold change ≥ 2), 285 (74 upregulated and 211 downregulated proteins), 120 (71 upregulated and 49 downregulated proteins), and 154 FGPs (60 upregulated and 94 downregulated proteins) are differentially expressed in the M, S, and F groups, respectively, compared to G group (Figure 5A). Moreover, some of these FGPs are exclusively detected in one group (Supplementary Data 1). Mostly, the differentially expressed FGPs in the M group are downregulated, whereas those in the S group are primarily upregulated. As for the F group, it has almost as many upregulated as downregulated differentially expressed FGPs. Overall, the volcano map and the normal distribution results illustrated in Figures 5B,C, respectively, indicated that the expression patterns of *B. thetaiotaomicron* FGPs varied significantly depending on the supplied polysaccharide diet. This is consistent with the Western blot results (Figure 2D), as well as with cluster analysis presented in Supplementary Figure 4.

**FIGURE 6:**
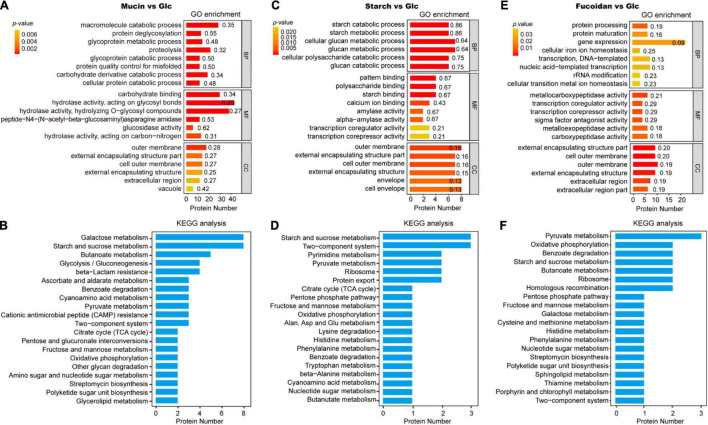
Gene ontology and Kyoto Encyclopedia of Genes and Genomes analysis of differentially expressed FGPs in different comparisons. GO and KEGG analysis of differentially expressed FGPs in mucin vs. glucose **(A,B)**, starch vs. glucose **(C,D)**, fucoidan vs. glucose **(E,F)**. The *p*-values were obtained based on Fisher’s exact test. The smaller the *p*-value, and the higher the significance level of the corresponding functional category enrichment. Numbers beside the bars mean rich factors, indicating the ratio of the number of differentially expressed proteins annotated to the function to the number of all proteins identified to the function.

The types and functions of the differentially expressed proteins in each group were analyzed by GO and KEGG enrichment analysis (Supplementary Data 2, 3). In group M, the FGPs are mostly downregulated, and the greatest change in expression is observed for Q8AB39 (SusC homolog) and Q8A4H4 (SusD homolog) proteins, both of which belong to the Sus protein family. Further, the most significantly upregulated FGPs in group M (more than 100 times) are Q8A2F5 (Chondroitin sulfate ABC lyase) and Q8A2I1 (Chondroitin sulfate ABC exolyase), both of which are chondroitin sulfate lyases (Supplementary Data 1). Based on the results of GO analysis (Figure 6A), the differentially expressed proteins are mainly outer membrane proteins that are involved in important metabolic biological processes and possess carbohydrate hydrolase activity. The KEGG analysis (Figure 6B) shows that galactose metabolism, starch and sucrose metabolism, butanoate metabolism and glycolysis are enhanced in the polysaccharide-treated samples, compared to the control.

In the S group, the greatest FGPs upregulation is observed for Sus-like proteins Q8A1G3 (Alpha-amylase SusG) and G8JZT0 (Outer membrane protein SusE). The levels of proteins associated with quorum sensing and bacterial secretion (e.g., Q89YY8, Preprotein translocase subunit YajC) are also substantially increased in the S group. In general, these proteins are implicated in the regulation of multidrug resistance, biofilm formation, extracapsular polysaccharide synthesis, and host-gut bacteria symbiosis (Freitas et al., 2003; Antunes et al., 2005; Pumbwe et al., 2008). The most downregulated FGPs expressions detected in group S are Q8A7S6 (Biotin carboxyl carrier protein) and Q89ZW1 (Deoxycytidylate deaminase). The GO analysis results presented in Figure 6C show that most differentially expressed proteins in group S are involved in important biological processes, especially the polysaccharide catabolic process. These proteins also have essential molecular functions, such as amylase activity and the binding of patterns, polysaccharides, starch, and calcium ions. As for the KEGG analysis results, they emphasize the role of the differentially expressed FGPs in vital physiological metabolism and signaling pathways such as starch and sucrose metabolism, two-component systems, pyrimidine metabolism, pyruvate metabolism, and ribosome and protein export, among others (Figure 6D).

The addition of fucoidan to the culture medium increases the expression of some *B. thetaiotaomicron* FGPs while decreasing the level of others. Notably, the most downregulated proteins are all efflux system proteins, such as Q89YN8 and Q89YN6. These FGPs play conservative roles in bacterial cationic efflux systems, particularly in quorum sensing, drug resistance, and survival competition, which can protect bacteria from toxic chemical components (Pumbwe et al., 2008; Wang et al., 2016; Hibberd et al., 2017; Ghotaslou et al., 2018). As for the upregulated proteins, the top three are Q8A347 (putative beta-xylosidase), Q8ABG9 (SusD homolog), and Q8AAC1 (ammonium transporter). Based on GO analysis, the differentially expressed proteins in the F group are mainly involved in gene expression, protein processing, transcription, DNA template formation, gene transcription, and protein mature expression. These proteins also exhibit metallocarboxypeptidase activity, transcription coregulator activity, sigma factor antagonist activity, and metalloexopeptidase activity, among other transcription-related molecular functions (Figure 6E). Finally, the KEGG analysis results indicate that the differentially expressed proteins are implicated in various metabolic processes of biological macromolecules (Figure 6F).

For the most part, the FGPs present different variation trends, depending on the nutritional conditions. However, eight FGPs were found to be consistently upregulated (in all groups), and 13 were regularly downregulated (Supplementary Data 4). The upregulated proteins were involved in some important metabolic pathways such as ribosome binding, starch and sucrose metabolism, butanoate metabolism, pyruvate metabolism, and two-component systems. As for the downregulated FGPs, they mainly participated in metabolic pathways related to gene expression and carbohydrate metabolism. Although the string results showed that most of the consistently changed proteins didn’t exhibit obvious direct protein-protein interactions (data not shown), the regular variation in the expression levels of these proteins may be related to the regulation of their functions and to the adaptation of *B. thetaiotaomicron* to polysaccharide nutritional conditions.

### Differentially Expressed Fucosylated Proteins With Nutrition-Sensitive Fucosylation Levels

**FIGURE 7:**
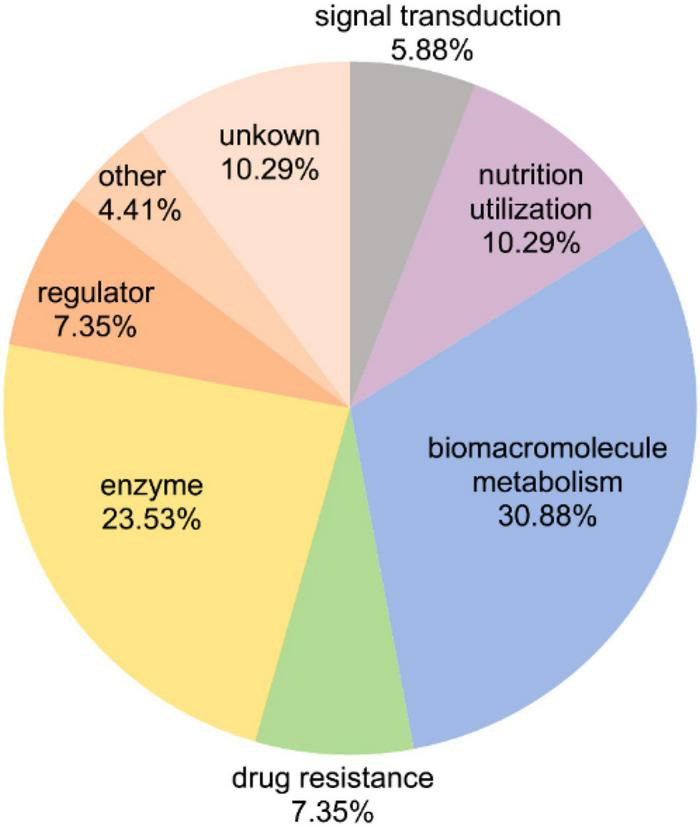
Functional classification of 65 key FGPs with significantly altered fucosylation level.

All above results showed the changes of the general FGPs expression levels. Notably, in addition to variations in protein expression levels, FGPs expressions were changed due to differences in the level of fucosylation. To identify the differentially expressed FGPs that were associated with altered fucosylation efficiency, quantitative global proteome analysis of *B. thetaiotaomicron* was also performed. Based on the obtained results (Supplementary Data 5), 65 key candidate FGPs were identified as being extremely sensitive to polysaccharide nutrition conditions (FGPs fold change/global protein fold change ≥2.0 or ≤0.5) (Table 1). They mainly involved in enzymatic activity, biomacromolecule metabolism, nutrition utilization, drug resistance and signal transduction (Figure 7).

**TABLE 1:** Key FGPs with significantly altered fucosylation level under different polysaccharide nutritional conditions.

	Accession	Protein name	Gene name	FGPs ratio	Global protein ratio	FGPs ratio/global protein ratio (≥2 or ≤0.5)
Mucin Vs. Glc	Q8ABF9	DUF2007 domain-containing protein	BT_0151	M unique	0.92	M unique
	Q8A6N6	Neutral zinc metallopeptidase	BT_1841	M unique	0.48	M unique
	Q8A1L5	Dihydropteroate synthase	BT_3646	M unique	0.99	M unique
	Q8AA80	Flavin reductase-like, FMN-binding	BT_0585	M unique	0.68	M unique
	Q8A0P2	Putative methyltransferase	BT_3979	M unique	1.00	M unique
	Q8A9W3	Chloramphenicol acetyltransferase (CAT-III)	BT_0702	M unique	0.98	M unique
	Q8A2F5	Chondroitin sulfate ABC lyase	BT_3350	141.31	N.F.	N.D.
	Q8A3A1	Putative cytochrome B subunit	BT_3053	2.34	0.45	5.17
	Q8A737	Biotin carboxyl carrier protein (BCCP)	BT_1688	2.06	0.70	2.97
	Q8AAC1	Ammonium transporter	BT_0544	10.52	4.05	2.60
	Q8A764	Two-component system sensor histidine kinase	BT_1661	2.71	1.32	2.05
	Q8A2D9	Glycoside transferase family 2	BT_3366	G unique	1.35	G unique
	Q8A2X2	DUF4884 domain-containing protein	BT_3183	G unique	1.53	G unique
	Q8A600	Choloylglycine hydrolase	BT_2086	G unique	1.39	G unique
	Q8A570	Transcriptional regulator	BT_2372	G unique	1.09	G unique
	Q8A1E2	Cationic outer membrane protein	BT_3724	G unique	1.21	G unique
	Q8A3J3	Putative lipase	BT_2961	G unique	1.02	G unique
	Q8AAW6	Ribose 5-phosphate isomerase B	BT_0346	G unique	1.40	G unique
	Q8AB01	Dihydrolipoamide acetyltransferase component of pyruvate dehydrogenase complex	BT_0311	G unique	1.08	G unique
	Q8A2B4	Ribonuclease H	BT_3391	G unique	1.40	G unique
	Q8A5K9	DNA polymerase III subunit alpha	BT_2230	0.48	4.18	0.12
	Q89ZS3	Glycogen debranching enzyme-related protein (4-alpha-glucanotransferase)	BT_4303	0.48	1.81	0.26
	Q8A846	DUF3078 domain-containing protein	BT_1328	0.28	1.00	0.28
	Q8A2J1	Thermostable beta-glucosidase B	BT_3314	0.35	1.01	0.35
	Q8ABG3	copper resistance protein NlpE	BT_0147	0.43	1.18	0.37
	Q8A4H0	Putative alpha-1,2-mannosidase	BT_2629	0.28	0.76	0.37
	Q8A730	Outer membrane efflux protein	BT_1695	0.32	0.72	0.45
	Q8A5I8	Putative membrane fusion protein	BT_2251	0.38	0.79	0.48
	Q8A4D7	Alpha-galactosidase	BT_2662	0.48	0.98	0.49
Starch Vs. Glc	Q8A5U4	Putative oxidoreductase	BT_2144	S unique	0.30	S unique
	Q8AAZ9	Flavodoxin	BT_0313	S unique	0.56	S unique
	Q8AA96	Putative acylhydrolase	BT_0569	S unique	0.40	S unique
	Q8A0Y6	Putative phosphohydrolase, Icc family	BT_3885	S unique	0.79	S unique
	G8JZS4	Glucan 1,4-alpha-glucosidase SusB	susB	8.90	1.18	7.51
	Q8AAC0	Nitrogen regulatory protein P-II	BT_0545	2.12	0.63	3.35
	Q8A2R5	SusC homolog	BT_3240	3.37	1.02	3.30
	Q8A6X0	Putative exported 24-amino acid repeat protein	BT_1755	2.37	0.79	3.01
	Q8AAC1	Ammonium transporter	BT_0544	9.60	3.93	2.44
	Q89YT0	Probable endonuclease 4	nfo	2.05	0.88	2.33
	Q8A0I6	HTH cro/C1-type domain-containing protein	BT_4035	G unique	2.02	G unique
	Q8AB01	Dihydrolipoamide acetyltransferase component of pyruvate dehydrogenase complex	BT_0311	G unique	1.10	G unique
	Q8A509	OMP_b-brl domain-containing protein	BT_2438	G unique	4.19	G unique
	Q8A582	Transcriptional regulator	BT_2360	G unique	2.46	G unique
	Q8A835	Undecaprenyl-phosphate alpha-N-acetylglucosaminyltransferase	BT_1339	G unique	2.18	G unique
	Q8A9K7	Lipoprotein signal peptidase	BT_0808	G unique	1.01	G unique
	Q8A7S6	Biotin carboxyl carrier protein	BT_1448	0.33	4.10	0.08
Fucoidan Vs. Glc	Q8A5U4	Putative oxidoreductase	BT_2144	F unique	1.06	F unique
	Q8A276	30S ribosomal protein S20	rpsT	F unique	0.86	F unique
	Q8A3F4	T5orf172 domain-containing protein	BT_3000	F unique	0.63	F unique
	Q8A7N3	DUF4465 domain-containing protein	BT_1491	F unique	0.57	F unique
	Q8A9X5	Two-component system response regulator	BT_0690	F unique	1.13	F unique
	Q8A9Z2	NADP(H) oxidoreductase	BT_0673	F unique	0.47	F unique
	Q8A295	UPF0145 protein	BT_3410	F unique	0.30	F unique
	Q8A0P2	Putative methyltransferase	BT_3979	F unique	0.63	F unique
	Q8A1N0	Uncharacterized protein	BT_3629	2.17	0.30	7.23
	Q8AAC0	Nitrogen regulatory protein P-II	BT_0545	3.64	0.91	4.01
	Q8A418	30S ribosomal protein S15	rpsO	2.02	0.83	2.44
	Q89ZC6	Putative MTA/SAH nucleosidase	BT_4451	G unique	1.36	G unique
	Q8A848	TPR domain-containing protein	BT_1326	G unique	1.16	G unique
	Q8AAW6	Ribose 5-phosphate isomerase B	BT_0346	G unique	0.90	G unique
	Q8A194	Ribosomal large subunit pseudouridine synthase D	BT_3772	G unique	1.85	G unique
	Q89YN8	Cation efflux system protein	BT_4693	0.03	0.62	0.04
	Q8A9Y6	Cation efflux system protein czcB	BT_0679	0.05	0.25	0.19
	Q8A9Y5	Cation efflux system protein czcA	BT_0680	0.41	1.37	0.30
	Q8A3H6	Transcriptional regulator	BT_2978	0.49	0.99	0.49

Overall, the addition of the three polysaccharides triggered a significant increase in the fucosylation levels of two-component system proteins and ammonium transporter proteins. In *B. thetaiotaomicron*, two-component systems (TCRS) are used to sense and degrade complex carbohydrates in the gut (Townsend, Raghavan, 2013). They are often co-localized with the genes encoding CAZymes and outer membrane sugar transporters so that they can co-regulate the utilization of polysaccharides (Xu et al., 2003; Raghavan and Groisman, 2010; Lynch and Sonnenburg, 2012; ,, and GroismanSchwalm, Townsend, and Groisman2017). However, the ammonium transporter proteins in *B. thetaiotaomicron* bacteria are involved in the absorption of nitrogen sources such as ammonia or ammonium (Luzhkov et al., 2006). A significant increase in the fucosylation levels of these two types of proteins is likely to affect their functions.

The FGPs related to chondroitin sulfate also show significant changes in fucosylation level under varying polysaccharide nutrition conditions. Particularly, the fucosylation levels of these FGPs are substantially increased (more than 100 times) upon the addition of mucin, which is consistent with the results presented above. Given that mucin cannot be degraded by the chondroitin sulfate ABC enzyme, it may significantly enhance the expression of this enzyme by acting as a signaling molecule that triggers a certain pathway in *Bacteroides*. Starch increases the fucosylation levels of Sus system proteins, which is possible to affect their ability to transport and utilize complex carbohydrates. In contrast, the fucosylation levels of efflux system proteins are substantially reduced by fucoidan. Other *B. thetaiotaomicron* FGPs showing changes in fucosylation levels upon the addition of different polysaccharides include those involved in polysaccharide metabolism, amino acid metabolism, short-chain fatty acid metabolism, folic acid biosynthesis, redox processes, RNA modification, and other important processes related to the growth and survival of *Bacteroides*. The above results show the fact that the fucosylation levels of proteins in *Bacteroides* are sensitive to various polysaccharide nutrition conditions, and the biological significance of this fact deserves to be further studied.

## Discussion

In this study, the MOE and CuAAC methods were successfully applied to the labeling and enrichment of *B. thetaiotaomicron* FGPs. To understand the effect of the supplied diet on FGPs expression patterns, *B. thetaiotaomicron* were cultured under different polysaccharide nutrition conditions, and then the levels of newly synthesized FGPs and global proteins were determined by label-free proteome analysis. As expected, corn starch, mucin, and fucoidan present different effects on the expression levels of the newly synthesized FGPs in *B. thetaiotaomicron*. In addition, the fucosylation levels of different FGPs also vary depending on the polysaccharide nutrition conditions. As a result, 65 key FGPs showing significant differences in fucosylation levels were screened. The biological functions of these proteins, including nutrition utilization, environmental response, and multidrug resistance, are also expected to be influenced by exogenous nutritional conditions. Ultimately, the altered biological functions will affect the colonization, survival, and growth of *B. thetaiotaomicron*. Herein, we determine that the proteins whose fucosylation levels are the most sensitive to nutrition conditions are chondroitin sulfate ABC enzyme, Sus proteins, and cationic efflux system proteins. Moreover, we show that corn starch, mucin, and fucoidan can significantly promote the fucosylation of the two-component system and ammonium transport proteins. To the best of our knowledge, these data have never been previously reported.

It has been proved that the surface glycoproteins of *B. fragilis* and *P. distasonis* can be labeled by FucAl and CuAAC (Besanceney-Webler et al., 2011). Here, *B. thetaiotaomicron*, a *Bacteroides* species that shares a common fucose acquisition system with all the other *Bacteroides* except for *P. distasonis*, was also successfully labeled by FucAl (Xu et al., 2003; Coyne et al., 2005). Based on the obtained results, the functions of differentially expressed FGPs in *B. thetaiotaomicron* are similar to those of *B. fragilis* glycoproteins with many proteins in the former bacterial species having additional important functions, such as carbohydrate metabolism (Fletcher et al., 2009, 2011). This indicates that FGPs are essential for different *Bacteroides* species. Overall, the experimental method applied herein was found to be sensitive, accurate, and robust.

Compared to other labeling relative quantification technologies, such as iTRAQ, TMT, and SILAC, label-free quantification is simpler. By quantitatively comparing the results of a large number of samples, the latter method ensures the authenticity and accuracy of the reported data. The method is also more suitable for the analysis of differentially expressed proteins in microorganisms kept under strict culture conditions (Nie et al., 2014; Anand et al., 2017; Velasquez et al., 2017; Wu et al., 2019). Over the years, the instruments and algorithms of label-free quantification have been significantly improved. Today, the accuracy of this method is comparable to that of SILAC (Cox et al., 2014; Valikangas et al., 2018). Thus, label-free quantification was used to investigate the effects of polysaccharide nutrition on the expression pattern of FGPs in *B. thetaiotaomicron*.

Recently, studies have shown that dietary fibers can be used to regulate the intestinal microbiome and to guide the production of short-chain fatty acids. Depending on their structure, these fibers exhibit different and highly specific effects on the microbiome composition that is related to selective bacterial adhesion and substrate utilization (Deehan et al., 2020). Therefore, it is hypothesized that polysaccharides with different structures and sources would affect the abundance and metabolism of *Bacteroide*s differently. Our results support this hypothesis. As key factors closely related to the growth and symbiosis of *Bacteroides*, as well as to substrate adhesion and nutrient utilization, FGPs and their fucosylation levels were found to be strongly influenced by the supplied polysaccharide diet. Moreover, most of the differentially expressed FGPs identified herein (especially CAZymes) are involved in macromolecular metabolic functions, such as polysaccharide and amino acid metabolism. These results indicate that different diets trigger different pathways of FGPs expression in *B. thetaiotaomicron* so that the bacteria may adapt to the environmental change. Compared with the results of global proteome, 65 key FGPs were selected and emphasized, which mainly involved in enzymatic activity, biomacromolecule metabolism, nutrition utilization, drug resistance and signal transduction. Changes of these proteins expression levels mainly came from the change of fucosylation levels, and reflected high sensitivity of their fucosylation to exogenous polysaccharide conditions. In contrast, some FGPs (such as Q8AAC1, Q8AB34) with significantly different expression levels were not selected, because their changes of protein expression level and changes of fucosylation level were similar or they were not found in global proteome. Therefore, sensitivity of fucosylation level to changes in exogenous nutritional conditions may play important roles in the growth, survival and colonization of *Bacteroides*.

*Bacteroides thetaiotaomicron* has an outstanding capability to degrade a wide range of polysaccharides including rhamnogalacturonan-II, the most structurally complex glycan known (Ndeh et al., 2017). Although the PULs for fucoidan has not been identified in *B. thetaiotaomicron*, this microbe encodes fucose cleavage and metabolism genes such as α-fucosidases (Sonnenburg et al., 2005) and several sulfatases genes (Ye et al., 2021), indicating its genetic potential for fucoidan degradation. So it’s reasonable to assume that during the 3-h incubation with *B. thetaiotaomicron*, the added polysaccharides were partially degraded and the released degradation products impacted cell metabolism. As a result, the proteome and protein fucosylation changed in *B. thetaiotaomicron*. Notably, it was possible that the fucose degraded from fucoidan or mucin could compete with FucAl for incorporation into newly synthesized proteins, which would lead to an underestimate of detectable protein fucosylation. To clarify to what extent the added polysaccharides are degraded, some analytical methods for sensitive determination and quantitation such as HPLC and multi angle light scattering (MALS) will be employed to monitor the degradation process in the follow-up work.

In this research, we just choose a relatively reasonable time point to study how the newly synthesized FGPs changes in response to the newly added polysaccharides. In the follow-up work, we will also explore the expression patterns of FGPs in *Bacteroides* at different time points after adding polysaccharide. Moreover, when the polysaccharide is the only carbon source, what changes of FGPs would make by the *Bacteroides* is an interesting question. To understand the reasons behind the changes in fucosylation levels and to assess the mechanisms of these changes, further biological experiments must be performed, including gene knockout and *in vivo* experiments. Given that the glycoproteins of most *Bacteroides* species have not yet been identified, the MOE and CuAAC methods must be applied to other *Bacteroides*. Overall, the results reported herein indicate that the combination of MOE and CuAAC is a suitable and effective strategy for the study of intestinal *Bacteroides* glycoproteomes, and that protein fucosylation is a potential key factor that may play a significant role in regulating multiple biological functions when *B. thetaiotaomicron* is subjected to changes in the nutritional environment.

## Data Availability Statement

The data presented in the study are deposited in the ProteomeXchange repository, accession number PXD023756.

## Author Contributions

HJ and GY conceived the project. XT, HF, and XW performed experiments. XT, HJ, and BC analyzed data. XT and HJ drafted the manuscript. XT, HJ, BC, and GY revised and approved the manuscript. All authors contributed to the article and approved the submitted version.

## Conflict of Interest

The authors declare that the research was conducted in the absence of any commercial or financial relationships that could be construed as a potential conflict of interest.

## Publisher’s Note

All claims expressed in this article are solely those of the authors and do not necessarily represent those of their affiliated organizations, or those of the publisher, the editors and the reviewers. Any product that may be evaluated in this article, or claim that may be made by its manufacturer, is not guaranteed or endorsed by the publisher.

## Footnotes

1
http://string-db.org/

## Abbreviations

*B. theaiotaomicron*: *Bacteroides theaiotaomicron*
FGPs: fucosylated glycoproteins
PULs: polysaccharide utilization site
Sus: starch utilization system
CAZymes: carbohydrate-active enzymes
AAL: Aleuria aurantia lectin
MOE: metabolic oligosaccharide engineering
CuAAC: Cu(I)-catalyzed azide-alkyne cycloaddition
AMM: anaerobic minimal medium
LC-MS: liquid chromatography mass spectrometry
FucAl: alkynyl-fucose
FDR: false discovery rate
GO: gene ontology
KEGG: Kyoto Encyclopedia of Genes and Genomes
SILAC: stable isotope labeling with amino acids.
