# Supplementary material for: Comparative Proteomic Analysis of Fucosylated Glycoproteins Produced by Bacteroides thetaiotaomicron Under Different Polysaccharide Nutrition Conditions
Source: Front Microbiol. 2022 Mar 4;13:826942. doi: 10.3389/fmicb.2022.826942 (PMC8931616; doi:10.3389/fmicb.2022.826942)
Supplement: Supplementary file 1 [file Data_Sheet_1.docx]

Supplementary Material

# Supplementary Data

**Supplemental Data 1:** Differentially expressed FGPs

**Supplemental Data 2:** GO analysis of Differentially expressed FGPs

**Supplemental Data 3:** KEGG analysis of Differentially expressed FGPs

**Supplemental Data 4:** Differentially FGPs with the same change trend of expression levels

**Supplemental Data 5:** Differentially expressed global proteins

# Supplementary Figures


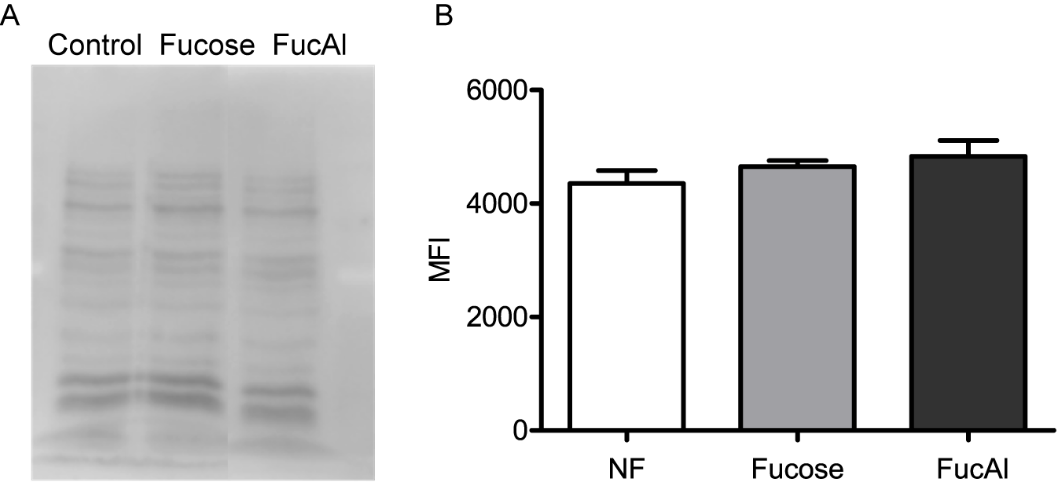


**Supplementary Figure 1.** **Effect of FucAl on the actual expression of FGPs in *B. thetaiotaomicron***

(A) Western blot analysis of the actual expression level of FGPs. *B. thetaiotaomicron* was incubated for 24 h with and without 200 μM FucAl, and 200 μM fucose. Cell lysates were prepared and analyzed by western blot with biotinylated Aleuria Aurantia Lectin(AAL) and HRP-anti-biotin antibody. (B) Flow cytometry analysis of the actual expression level of FGPs on the cell surface of B. thetaiotaomicron. B. thetaiotaomicron was incubated with and without 200 μM FucAl, and 200 μM Fucose for 24 h. Cells were collected and analyzed by flow cytometry with biotinylated Aleuria Aurantia Lectin(AAL) and Streptavidin Alexa Fluor™ 488 conjugate.


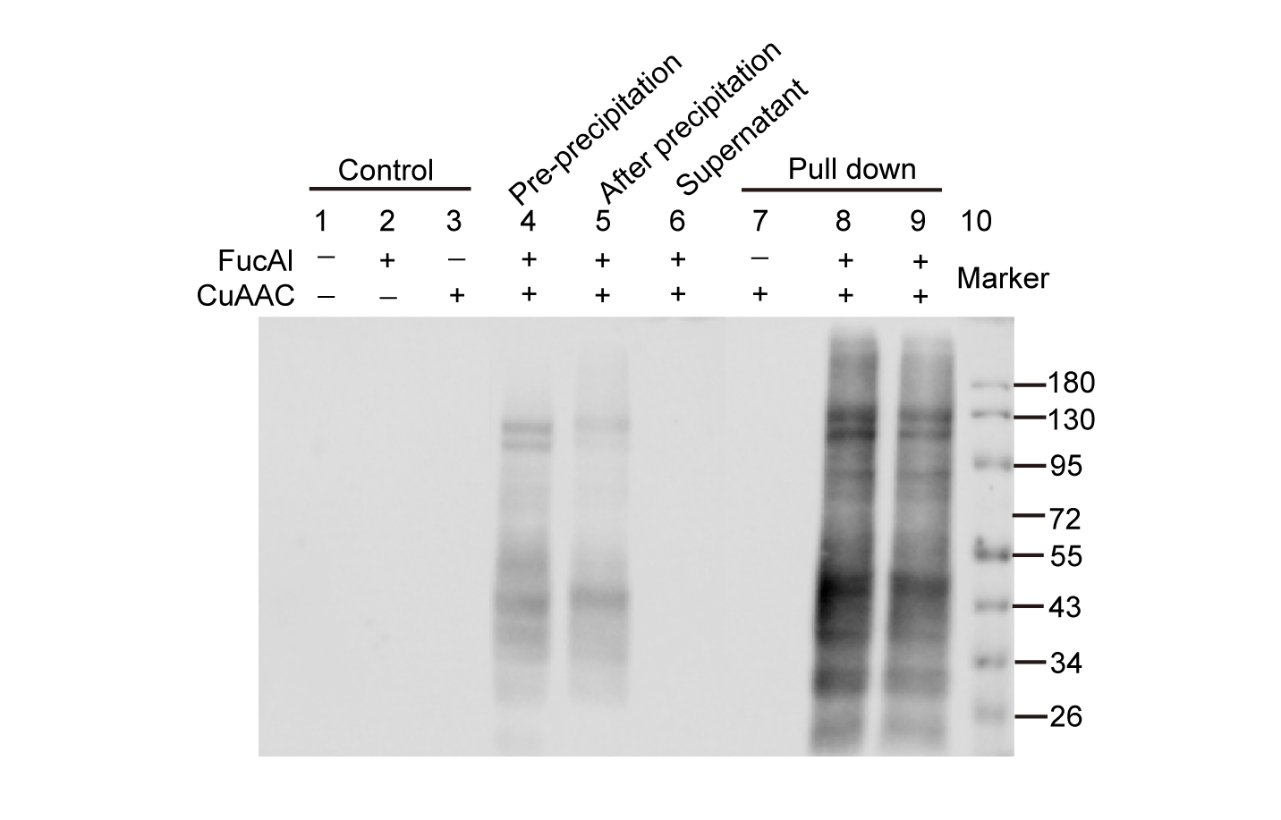


**Supplementary Figure 2. Detection of the streptavidin enrichment of alkyne-modified proteins by Western blot**

Samples treated with FucAl either CuAAC were as control (lanes 1–3, 7). As for experimental group (lanes 4, 5, 8, 9), B. thetaiotaomicron were cultured in the presence of 200 μM FucAl, lysed, reacted with biotin-azide via CuAAC(lane 4), then precipitated by methanol and then re-dissolved (lane 5). After that, alkyne-modified proteins were enriched by streptavidin beads, and the supernatant (lane 6) after enrichment were detected. Alkyne-modified proteins (lane 8, 9) bounded to the streptavidin beads were released by heating and detected by Western blot with HRP-anti-biotin (1:10000 dilution).

**
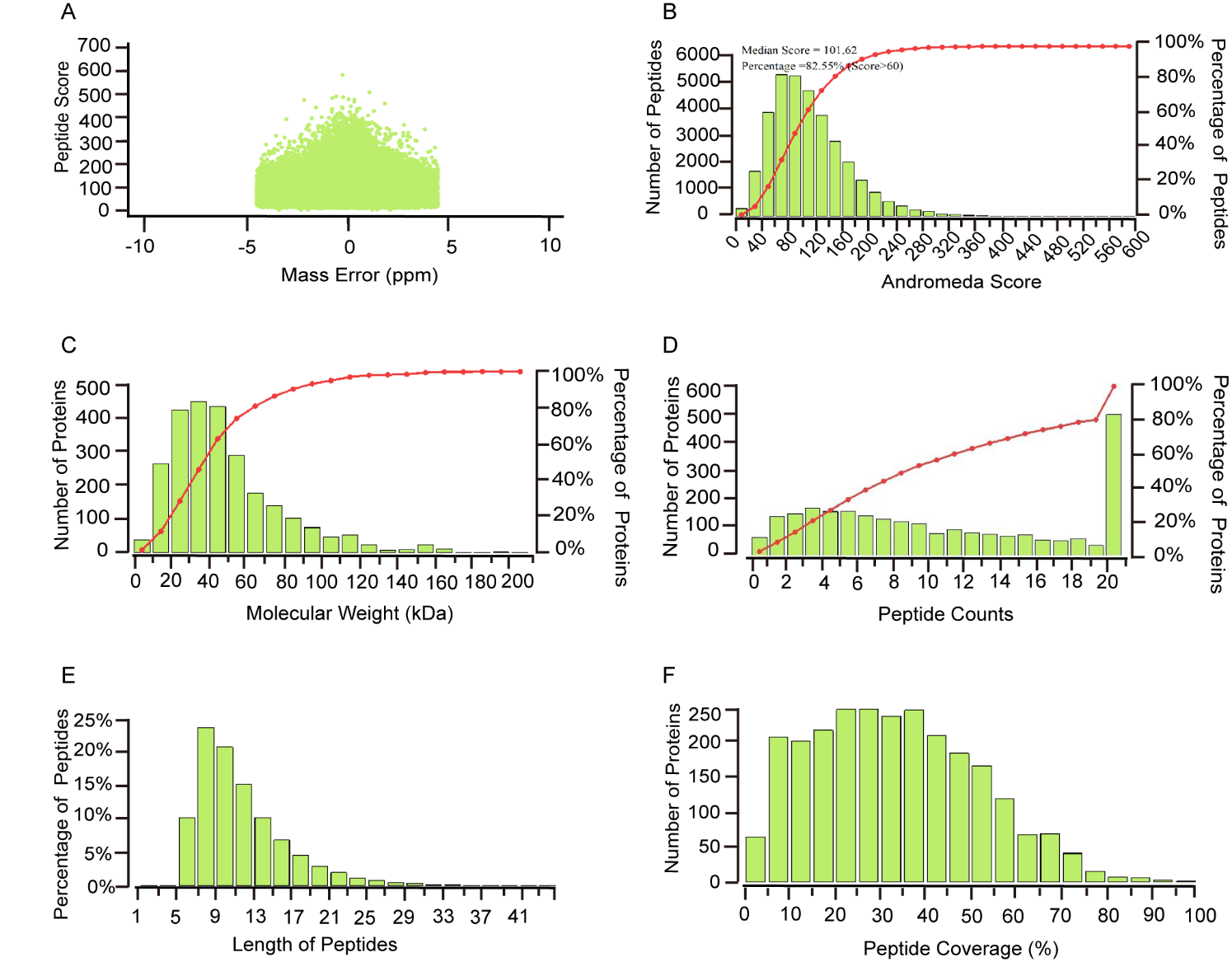
**

**Supplementary Figure 3. Quality evaluation of differentially expressed FGPs identified by label-free quantitative.**

(A) Peptide ion mass deviation distribution of identified peptides depicting peptide ion mass deviation of theoretical mass-to-charge ratio between mass-to-charge ratio measured by mass spectrometry (ppm) (x axis), and the MASCOT peptide score (y axis). (B) Peptide ion score distribution depicting the Andromeda peptide score (x axis), number of peptides (left y axis), the cumulative percentage of peptides not higher than the corresponding ion score (right y axis). (C) Molucular weight distribution (left y axis) and the cumulative percentage of proteins (right y axis) of iendified FGPs. (D) Number of identified peptides distribution depicting the peptide counts (x axis), number of proteins (left y axis), the cumulative percentage of proteins not higher than the corresponding peptide counts (right y axis). (E) Length of peptides distribution of identified peptides. (F) Peptides coverage distribution of identified proteins.


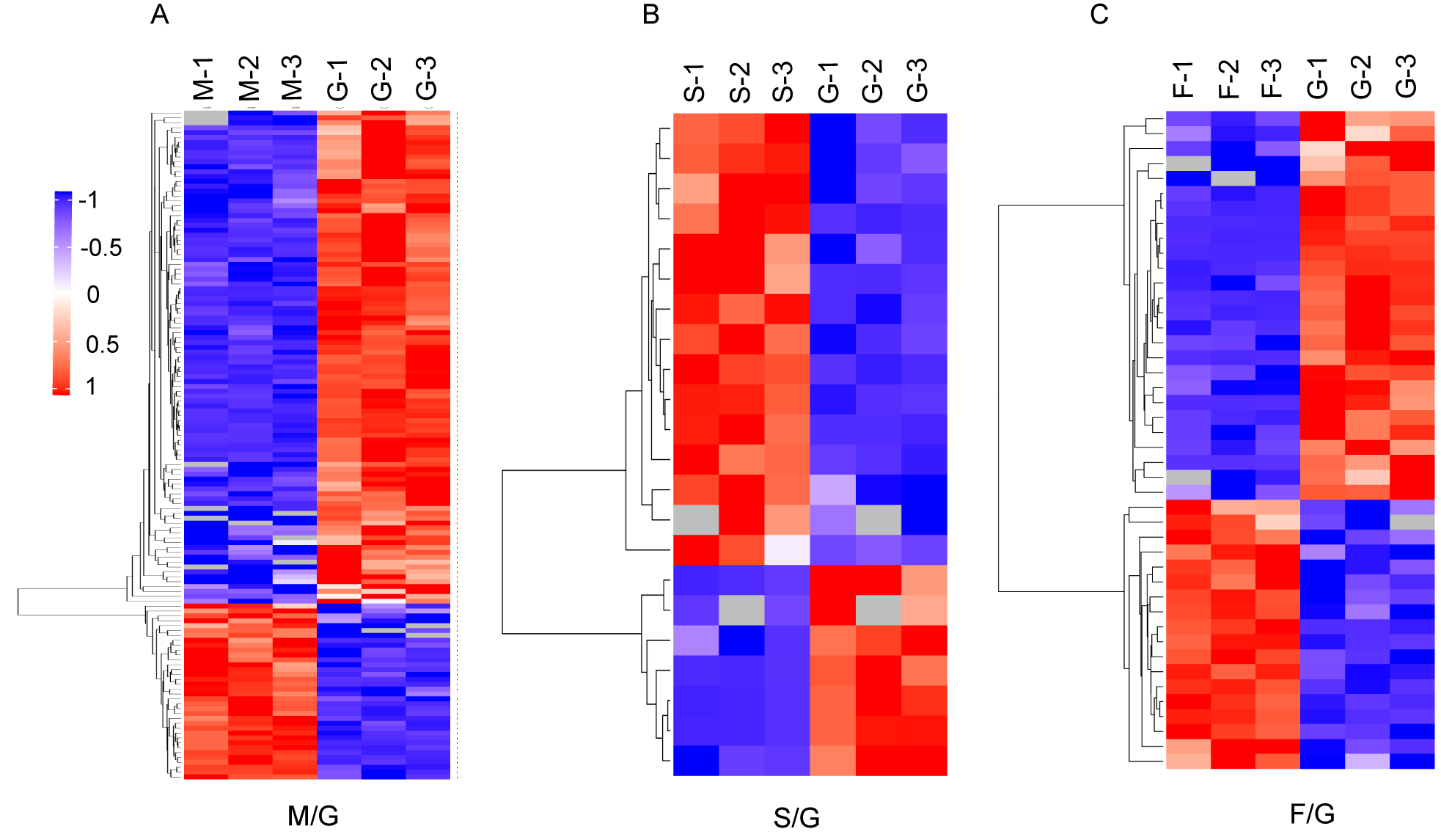


**Supplementary Figure 4. Hierarchical cluster analysis of differentially expressed FGPs**

Hierarchical cluster analysis of differentially expressed FGPs in mucin vs glucose (A), starch vs glucose (B) and fucoidan vs glucose (C). Each row represents a significantly differentially expressed protein, and each column represents a sample. Log2(expression level) values of significantly differentially expressed proteins are shown in the heat map in different colors. Red, up-regulated protein; blue, down-regulated protein; gray, no protein quantitative information.
